# Psychological impact of an acute intervention on medical-psychological emergency unit professionals: the example of hurricane Irma

**DOI:** 10.1192/bjo.2021.61

**Published:** 2021-06-14

**Authors:** Oriane Razakarivony, Nagham Khanafer, Jean-Marc Philippe, Nathalie Prieto

**Affiliations:** Cellule d'Urgence Médico-Psychologique, Hôpital Edouard Herriot, Hospices Civils de Lyon, France; and Faculty of Medicine, Lyon 1 University, France; Unité d'hygiène, épidémiologie et prévention, Hôpital Edouard Herriot, Hospices Civils de Lyon, France; and Public Health, Epidemiology and Evolutionary Ecology of Infectious Diseases (PHE^3^ID), Université de Lyon, France; Direction Générale de la Santé, Ministère de la Santé, France; Cellule d'Urgence Médico-Psychologique, Hôpital Edouard Herriot, Hospices Civils de Lyon, France; and Centre Régional du Psychotraumatisme, Hospices Civils de Lyon, France

**Keywords:** Burnout, compassion satisfaction, ProQOL, psychiatric, traumatic stress

## Abstract

**Background:**

Medical-psychological emergency units (Cellules d'Urgence Médico-Psychologiques, CUMP) are deployed following major events where there is a risk of psychological trauma, in order to provide acute and proper psychological care for the victims.

**Aims:**

To describe and evaluate the risk of a psychological impact on CUMP professionals after their participation in the aftermath of the hurricane Irma natural disaster. CUMP teams consist of medical and paramedical staff, who can have permanent or volunteer status. We reasoned that there might be a psychological and emotional impact on CUMP professionals, despite their own expertise in the field, after their intervention following hurricane Irma.

**Method:**

A cross-sectional survey was conducted during a feedback meeting. Participating professionals completed a sociodemographic questionnaire and the Professional Quality of Life (ProQOL) scale (5th French version), which is composed of three subscales: compassion satisfaction, burnout and secondary traumatic stress (STS).

**Results:**

A total of 53 participants were included with 24 (45.3%) psychiatrists, 15 (28.3%) paramedical staff and 14 (26.4%) psychologists. The median age was 46 years (range 39–55.5) and 29 (54.7%) were women. We found that psychiatrists compared with other professions had higher secondary traumatic stress scores (*P* = 0.007) and that volunteer psychiatrists had higher burnout scores than permanent psychiatrists (*P* = 0.03).

**Conclusions:**

These preliminary results suggest a psychological impact attributable to leadership status, which was reserved for psychiatrists. The results also underline the need for a supportive accompaniment for such teams by promoting formation improvement, psychological support and team cohesion.

## Background

French medical-psychological emergency units (Cellules d'Urgence Médico-Psychologiques, CUMP) are deployed during major events where there is a risk of psychological trauma such as natural disasters or terrorist attacks in order to provide acute and appropriate psychological care for the victims. CUMP professionals have permanent or volunteer status. Being a permanent member implies daily and routine work within the same team in the CUMP facilities and being spread out for acute events still within the same team. Whereas volunteer professionals usually work in another mental health department on a daily basis but have specific training in trauma interventions. They are requisitioned as CUMP staff only for acute events. Their day-to-day activity therefore differs from their activity during a crisis intervention.^1^

To our knowledge, the psychological impact of acute intervention has been well studied in rescuers such as firefighters or emergency mobile services (EMS)^2–6^ but not in the specific population of CUMP professionals who are confronted to psychological distress exclusively. Although they do not have to deal with life-threatening emergencies, they must always be prepared to deal with extreme and stressful situations, just like firefighters or EMS. Permanent uncertainty, instability and extreme work conditions may have a psychological impact even on these well-trained professionals. Nevertheless, these CUMP teams may particularly need to go beyond uncertain and unfamiliar conditions. They have to be ready to cope with weighty emotional charge during the mission and to be sufficiently resilient to take care of people needing their help.^7–11^ We think that, despite their own expertise in the area of psychology and psychiatry, there is a psychological and emotional impact on CUMP professionals because of their work relating to acute psychological intervention.

## Aims

In this context, we undertook a study to evaluate the psychological state of CUMP professionals who had participated in providing acute psychological support after a natural disaster linked to the category-five hurricane Irma. Irma hit the French Antilles and mainly the islands of Saint-Martin and Saint-Barthélémy on 6 September 2017. This hurricane provoked major damage to buildings, water and electricity networks, and sanitation. Conditions on site were particularly disastrous. Moreover, Irma was followed on 18 September 2017 by a second category-five hurricane Maria, which mainly affected the north of Martinique and the south of Guadeloupe.^12^

## Method

### Study population

A cross-sectional descriptive study was conducted among CUMP professionals involved in providing acute psychological interventions following hurricane Irma. One hundred CUMP professionals were involved in the management of this disaster and one-quarter of them were from the French Antilles. Professionals living in Metropolitan France were invited to participate in a feedback meeting. Participation in this meeting was essential as it consisted of feedback on the crisis intervention following the Irma disaster, allowing the re-immersion of the participants. The inclusion criteria were: (a) participants who completed the two self-administered, anonymous questionnaires by the end of the meeting, and (b) people not recently mobilised for another natural catastrophe. The rationale for the last criteria was to avoid having findings that related to different events that could also have contributed to the development of secondary traumatic stress and burnout.

According to French law, such a study does not require ethics committee approval and was approved by the Hospital Institutional Review Board. All participants provided written consent.

### Data collection

A feedback meeting was held in Lyon, France on 9 January 2018. All participants (*n* = 69) were informed about the opportunity to take part in this study, which involved completing two self-administered, anonymous questionnaires by the end of meeting. A total of *n* = 54 participants answered the survey.

A demographic profile sheet contained the following variables: age, gender, core profession, type of engagement in CUMP (permanent staff or volunteer), year of engagement in CUMP, location and duration of intervention.

The second part of the survey concerned the Professional Quality of Life (ProQOL) Scale, version 5.^13^

The ProQOL is not a diagnosis scale, rather a screening tool. It has been used to measure compassion satisfaction, burnout and secondary traumatic stress levels in respondents. ProQOL consists of a 30-item Likert scale ranging from 1 (never) to 5 (very often) and integrates three subscales: compassion satisfaction, burnout and secondary traumatic stress. The ProQOL asks participants to rate how frequently they experienced some feelings relating to their work as helpers in the past 30 days.^13^

The calculation of scores was achieved as following:

- reversing score items 1, 4, 15, 17 and 29;
- adding the items by subscale; and
- converting the raw score to a *t*-score.

Raw scores, available for each category, may range from 10 to 50, and the total percentiles available are 100. A raw score of 22 or below indicates low levels of compassion satisfaction, burnout and secondary traumatic stress; scores from 23 to 41 suggest average levels; and scores above 41 mean a high level.

A higher score on the compassion satisfaction scale means higher satisfaction associated with the ability to be an effective caregiver in one's work. A high risk of burnout is considered when the score for burnout is ≥57. Finally, higher scores on the secondary traumatic stress scale mean distress related to secondary exposure to extreme or traumatic work-related events.^13^

### Statistical analysis

The characteristics of participants were compared. The distribution of continuous variables was checked. The outcome of the participants regarding their quality of life post-intervention was reported by raw and *t*-scores. To assess differences between subgroups, continuous variables were expressed by mean (range) or median (interquartile range, IQR) values and compared using the Kruskal–Wallis test or Mann–Whitney *U-*test. Categorical variables were presented as proportions and compared using the χ^2^-test and the Fisher exact test. To evaluate the mean of duration of stay in the Islands and the quality of life of participants after the end of intervention we undertook Kaplan–Meier analysis with the log-rank test. Two-tailed *P*<0.05 values were considered statistically significant in all tests. Statistical analyses were undertaken with the statistical software package for the social sciences (version 17.0 for Windows, SPSS, Inc., Chicago, IL).

## Results

### Description of the study population

A total of 69 professionals attended the feedback meeting and 54 (78%) of them filled out the questionnaire. One participant did not complete the demographic data and was excluded from the final analysis.

There were 29 women (54.7%) and 24 (45.3%) men (gender ratio female/male was 1.2). The median age was 46 (39–55.5) years. There were 16 (30.2%) permanent professionals and 37 (69.8%) volunteers. There were more psychiatrists (*n* = 24, 45.3%) than paramedical staff (*n* = 15, 28.3%) or psychologists (*n* = 14, 26.4%). Data on years of experience was completed by 51 participants with a median of 9 (3–14) years.

The location of the intervention was Saint-Martin (*n* = 15, 28.3%), Saint Barthelemy (*n* = 15, 28.3%) and other locations (Guadeloupe, Martinique, Airport reception) (*n* = 23, 43.4%). Arrival time after hurricane Irma was reported by 49 participants: 17 (34.7%) arrived within 7 days, 14 (28.6%) arrived between 8 and 14 days after the hurricane and 18 (36.7%) arrived after 15 days. The duration of stay of half of them was 9 (range 8–10) days.

### ProQOL scores analysis

The raw and *t*-score of compassion satisfaction, burnout, and secondary traumatic stress are summarised in Table 1. The scores were not calculated for a few participants who did not respond to some items. Nine participants did not respond to the item 22 ‘I believe I can make a difference through my work’ and we hypothetize that it was not fully understood by the participants. Therefore, statistical analysis was undertaken as follows: compassion satisfaction (*n* = 41), burnout (*n* = 50) and secondary traumatic stress (*n* = 51).

**Table 1:** Description of raw and *t*-scores of compassion satisfaction, burnout and secondary traumatic stress in the study population

	*n* (%)	Mean	Minimum	Maximum
*Raw score*				
Compassion satisfaction, *n* = 41		41.5	33	50
Low ≤ 22	0 (0)			
Average 23–41	20 (48.8)			
High ≥ 42	21 (51.2)			
Burnout, *n* = 50		19.7	13	31
Low ≤ 22	36 (72)			
Average 23–41	14 (28)			
High ≥ 42	0 (0)			
Secondary traumatic stress, *n* = 51		18.2	12	26
Low ≤ 22	45 (88.2)			
Average 23–41	6 (11.8)			
High ≥ 42	0 (0)			
*T-score*				
Compassion satisfaction, *n* = 41		50	28.9	71.0
Low ≤ 44	14 (34.1)			
Average 45–56	16 (39)			
High ≥ 57	11 (26.8)			
Burnout, *n* = 50		50	33.5	77.7
Low ≤ 43	19 (38)			
Average 44–55	17 (34)			
High ≥ 56	14 (28)			
Secondary traumatic stress, *n* = 51		50	32.8	71.8
Low ≤ 42	13 (25.5)			
Average 43–55	25 (49)			
High ≥ 56	13 (25.5)			

The severity range for the three scores was not reached for any subgroup. A higher score of the compassion satisfaction scale means higher satisfaction associated with the ability to be an effective caregiver in one’s work. A high risk of burnout is considered when the score of burnout equals 56 or higher. Finally, higher score of secondary traumatic stress scale means distress related to secondary exposure to extreme or traumatic, work-related events. The differences regarding compassion satisfaction, burnout and secondary traumatic stress scores were not statically significant regarding age, gender, professional category, CUMP type of engagement or conditions of intervention.

However, the comparison between psychiatrist and other professions showed significantly higher secondary traumatic stress mean raw and *t*-score in psychiatrics (*P* = 0.007) (Table 2).

**Table 2:** Comparison of raw and *t*-score for the three subscales between psychiatrists and the other participating professionals

	Psychiatrics, *n* = 24			Other professionals, *n* = 29			
	Mean	Minimum	Maximum	Mean	Minimum	Maximum	*P*
Raw score							
Compassion satisfaction, *n* = 41	41.2	33	49	41.9	37	50	0.72
Burnout, *n* = 50	20.5	15	31	19.0	13	26	0.23
Secondary traumatic stress, *n* = 51	19.5	13	25	17.0	12	26	0.007
*T*-score							
Compassion satisfaction, *n* = 41	49.1	28.9	68.5	50.9	38.8	71.0	0.72
Burnout, *n* = 50	51.8	38.4	77.7	48.3	33.5	65.4	0.23
Secondary traumatic stress, *n* = 51	53.8	35.6	69.0	46.7	32⋅8	71.8	0.007

Moreover, the comparison between volunteer and permanent psychiatrists revealed that the mean burnout raw and *t*-scores were significantly higher in volunteer clinicians (*P* = 0.03). The results are summarised in Table 3. No significant difference was observed between volunteer and permanent staff among the other professions.

**Table 3:** Comparison of raw and *t*-scores for the three subscales between permanent and volunteer psychiatrists

	Volunteer, *n* = 14	Permanent, *n* = 10	*P*
*Raw scores*			
Compassion satisfaction			
Mean (minimum–maximum)	40.3 (33–48)	42.3 (38–49)	0.32
Level of score, *n* (%)			0.26
≤22	0 (0)	0 (0)	
23–41	7 (58.3)	3 (33.3)	
≥42	5 (41.7)	6 (66.7)	
Burnout			
Mean (minimum–maximum)	22.1 (16–31)	18.2 (15–24)	0.03
Level of score, *n* (%)			0.04
≤22	7 (50)	9 (90)	
23–41	7 (50)	1 (10)	
≥42	0 (0)	0 (0)	
Secondary traumatic stress			
Mean (minimum–maximum)	20.4 (14–25)	18.3 (13–23)	0.07
Level of score, *n* (%)			0.46
≤22	11 (78.6)	9 (90)	
23–41	3 (21.4)	1 (10)	
≥42	0 (0)	0 (0)	
*T-scores*			
Compassion satisfaction			
Mean (minimum–maximum)	47.0 (28.9–66.0)	52.0 (41.2–68.5)	0.32
Level of score, *n* (%)			0.90
≤44	5 (41.7)	3 (33.3)	
45–56	4 (33⋅3)	3 (33.3)	
≥57	3 (25)	3 (33.3)	
Burnout			
Mean (min-max)	55.8 (40.9–77.7)	46.3 (38.4–60.5)	0.03
Level of score, *n* (%)			0.12
≤43	3 (21.4)	4 (40)	
44–55	4 (28.6)	5 (50)	
≥56	7 (50)	1 (10)	
Secondary traumatic stress			
Mean (minimum–maximum)	56.2 (38.4–69.0)	50⋅4 (35⋅6–63⋅5)	0.07
Level of score, *n* (%)			0.06
≤42	1 (7.1)	2 (20)	
43–55	5 (35.7)	7 (70)	
≥56	8 (57.1)	1 (10)	

### Kaplan–Meier analysis

**Fig. 1:**
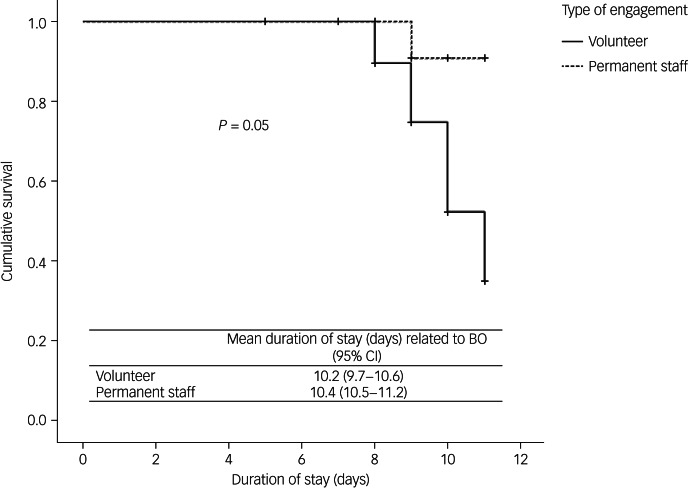
Kaplan–Meier curve for the development of burnout (BO) related to the duration of intervention by type of engagement in the Cellules d'Urgence Médico-Psychologiques (CUMP).

Of the 53 participants, 31 (58.5%) reported burnout *t*-score above 44. Among them, 17 were psychiatrics (54.8%). The analysis according to the type of engagement, showed that in psychiatrist group, the volunteers were more at risk of burnout than permanent staff (trend, *P* = 0.05) (Fig. 1). Stratified analysis showed that the association between the type of professional engagement and burnout according to the duration of intervention was only observed in psychiatrics (*P* = 0.05 *v*. *P* = 0.20 in non-medical participants).

## Discussion

### Main findings

The results of this study showed no significant alteration in ProQOL scores for most professional categories. However, psychiatrists seemed more prone to have higher secondary traumatic stress scores compared with other professionals. In addition, burnout scores were higher in volunteer psychiatrists than in those with permanent status. Recent reviews have shown that mental health professionals and psychiatrists are particularly at risk of emotional exhaustion and burnout.^14–16^ A recent meta-analysis on the risk factors for secondary traumatic stress in trauma-therapeutic workers found that case-load volume and frequency, personal history of trauma and work or social support may be implicated.^17^

Working conditions in disasters, with inadequate ergonomics and high time pressure may, among other issues, play a role in the generation of compassion-fatigue when providing acute psychological interventions.^18^ Acute CUMP intervention work combines several of these factors, particularly in terms of work overload and stressful working conditions. In addition, feelings of isolation and lack of support can partially explain the higher secondary traumatic stress scores in psychiatrists. Indeed, they need to deal with increased responsibility and provide acute fast responses as they take on the role of chief in the team during crisis interventions such as hurricane Irma.^1^

The higher score for burnout in volunteer psychiatrists may be, in part, explained by their working conditions. They may have been overwhelmed by the increased responsibilities they had to carry out with an unfamiliar team and in chaotic conditions. Furthermore, as physicians, volunteer psychiatrists were recruited as crisis team leaders for an acute intervention even if they were not used to this role. Moreover, it has been suggested that the risk of burnout can be reduced when strong relationships and high levels of trust are present between team members.^18^ In comparison, permanent psychiatrists are typically working with their usual team and this may explain why the burnout scores were lower compared with their volunteer colleagues. Further studies should be undertaken in order to explore these hypotheses.^4^

### Strengths and limitations

To the best of our knowledge, this is the first study that has focused on the impact that working on a stressful acute intervention has on the specialised psychological CUMP members participating in providing support. Nonetheless, some limitations should be acknowledged. First, the cross-sectional design does not allow us to draw direct causality between the variables. Thus, the results of our analyses remain exploratory with multiple comparisons and so we cannot exclude the possibility that our results emerge from unknown confounding factors. Moreover, results were observed in a small sample which do not allow for any generalization. We assumed that the results of our study must be stated with all possible precautions, and larger studies (and/or many studies with a similar design) are needed to confirm what we observed in this one. Higher burnout scores in volunteer psychiatrists may independently come from their day-to-day mental health activity rather than their acute intervention regarding hurricane Irma. Prospective studies are required to confirm or to refute these results.

Second, the study was conducted in a specific population and the results cannot be extrapolated to other mental healthcare professionals. Further studies comparing the CUMP team and other mental health professionals could be of great interest. Third, the use of a self-questionnaire rather than a more objective assessment could be considered as a limitation. Self-report measures may be influenced by recall bias.

Finally, the construct validity of the ProQOL and the way of analysing the subscales may be unclear, as discussed in other studies.^19,20^ In addition, the use of cut-off score categories may have been overly inclusive and have favoured a false-positive.^13^ This may have an impact on the interpretation of the results. However, we can consider this a relatively good construct validity with the growing number of publications using ProQOL. Misunderstanding about certain items such as item 22 also raises questions about potential inaccuracy in the responses and the need for more explanations about the measure for participants.

### Implications

This study suggests that psychiatrists seem more at risk of secondary traumatic stress and burnout than other professions, such as psychologists and paramedical staff, after participating in an acute psychological intervention. These preliminary results should be confirmed by large prospective studies. Nevertheless, our findings may reflect the probability that some psychiatrists may be more responsive in a psychological crisis intervention as they are required to take on a leader position, including increased responsibility and decision-making in very stressful conditions. This study could encourage greater care in the selection and training of team leaders and in the management of teams operating in such hostile environments. It also seems important to ensure a structured organisation which allows volunteer psychiatrists to benefit from a pre-intervention briefing with the possibility of calling for support from an experienced psychiatrist if needed. An efficient formation, better psychological support and a good team cohesion should be promptly and adequately implemented in daily practice, especially in the volunteer staff group.

## Data Availability

The data that support the findings of this study are available from the corresponding author (NK), upon reasonable request.
